# The effect of pregnancy on maternal cognition

**DOI:** 10.1038/s41598-021-91504-9

**Published:** 2021-06-09

**Authors:** Giulia Barda, Yossi Mizrachi, Irina Borokchovich, Lampl Yair, Diana Paleacu Kertesz, Ron Dabby

**Affiliations:** 1 Sackler Faculty of Medicine, Tel-Aviv University, Tel Aviv, Israel; 2 Division of Maternal Fetal Medicine, Department of Obstetrics and Gynecology, Edith Wolfson Medical Center, Holon, Israel; 3 Department of Neurology, Edith Wolfson Medical Center, Holon, Israel; 4 Neurology Service and Memory Clinic, Abarbanel Mental Health Center, Bat Yam, Israel

**Keywords:** Neuroscience, Medical research, Neurology

## Abstract

To determine whether there are differences in measures of cognitive function between second and third trimester pregnant women compared to non-pregnant controls. This prospective study comprised 40 pregnant and 40 non-pregnant women, 20–40 years old, native-Hebrew speakers who were recruited from the outpatient clinics during a period of nearly 2 years. The patients underwent cognitive and affective evaluation. The performance on the three following tests: difficult and total items of Verbal Paired Associates, the Digit Span—forward and the Naming Objects and Fingers test scores were significantly better among non- pregnant women. All the other test results were similar between the two groups, including the depression scores. On multivariate linear regression analysis, after adjusting for age and years of education , Verbal Paired Associates total score (*p* = 0.04), and Naming Objects and Fingers (*p* = 0.01) remained significantly associated with pregnancy, but not Digit Span (*p* = 0.09). Our study demonstrates an impairment in memory among pregnant women. Furthermore language skills, particularly naming, were also impaired, a finding which has not been previously described.

## Introduction

Normal pregnancy is characterized by elevated levels of several plasma steroid hormones^1–4^, which are known to cross the blood–brain barrier^5^. Estrogen has a role in neuroendocrine regulation and reproductive behaviors. Furthermore, it has a neuroprotective effect on degenerative disease or injury^6^. Studies based upon animal models reported better spatial learning and memory during pregnancy in rats^7,8^. Human studies report that hormonal changes affect cognitive performance. Therefore, the change in hormone levels during pregnancy provides a unique opportunity to study their effect on cognitive functions^9–20^. Furthermore the latest literature report structural brain changes during pregnancy and early postpartum which may confer a protective effect against the aging process and might be related to better cognitive outcomes persisting in older age^21,22^.

The current literature related to cognitive changes during pregnancy is controversial. Most studies show a deficit in cognitive functioning during pregnancy^12,15,20,23–28^, while others report an improved performance^29^, or no effect at all^9^. Poor concentration, memory deficit, disorientation and executive function impairment were all reported in previous studies. Nevertheless most data report on memory and concentration decline during pregnancy, while data regarding language abilities are scarce.

This study was designed to investigate differences in measures of cognitive function related to attention, memory, and learning. We also aimed to add information regarding language abilities of second and third trimester pregnant women and non-pregnant controls. For this purpose we used a complex structured cognitive battery including multiple tasks for multiple cognitive functions, in order to detect possible changes in other functions as well.

## Materials and methods

This was a prospective cohort study. The study comprised 80 participants recruited from our obstetric outpatient clinic, and included 40 pregnant and 40 non-pregnant women. Inclusion criteria for all the women recruited: age between 20 and 40 years old and native Hebrew speakers, born and educated in Israel. The majority were of Jewish origin (there were two Arab Muslim women in the pregnant group and three in the non-pregnant group). Exclusion criteria included a history of learning and/or attention and/or hyperactivity disorder (ADHD), any psychiatric disorder, brain injury, epilepsy, multiple sclerosis, mental retardation or dementia, any other neurological disorder known to be accompanied by cognitive decline, and any history or current substance abuse. Pregnant participants were in the second or third trimester. Non-pregnant women came to the obstetric clinic for pre pregnancy consultation and were not recruited if they had given birth during the preceding year.

The study was approved by the E.Wolfson Institutional Review Board (Reference No. 0216-16 WOMC). All experiments were performed in accordance with relevant guidelines/regulations. All participants signed an informed consent.

Participants were recruited through the physician assistance office between January 2017 and October 2018 and underwent the following tests, which were adapted from the ADAS-COG scale^30^. It has been proven that the ADAS-Cog is a reliable tool for detecting and studying patients with mild cognitive impairment. The results also indicated that demographic variables such as age and education do not play a significant role in the diagnosis of mild cognitive impaired patients based on the ADAS-Cog scores. In the ADAS-Cog scale the items are weighted and have their categories hierarchically ordered. Thus we chose it over other self-reported scales like “questionnaire on demographic, health, and sleep details, the Inventory of Memory Experiences, and tests of implicit, explicit, and working memory”^31^.

Verbal Paired Associates test: a test of verbal and learning memory. Participants were asked to remember words that were paired and read aloud to them, including four easy and four difficult pairings. We performed three trials of eight word pairings with scores of one point for every correct answer and zero for an incorrect association. The maximum score for the three trials was 12 for the easy and 12 for the difficult associations.

### Digit span

A test of working memory consisting of two trials forwards and two trials backwards. Each consisted of six consecutive items in increasing order from three to eight digits. After reading the digits, the participants were asked to repeat the sequence. The test was discontinued after failure on two consecutive trials of any item, whether forwards or backwards. We awarded two points if the participant passed both trials, one for only one successful trial, and zero if both trials failed. Maximum score: 12.

### Trail making test (TMT)

A test that measures the speed and accuracy of motor response. In part A of this test, the participant was asked to draw a line connecting 25 numbers in ascending order. Part B required drawing a line to connect alternating numbers and letters in an ascending pattern. The time needed to complete the task was measured. If an error was made, we pointed it out immediately and allowed the participant to correct the mistake. The correction of errors was included in the total completion time for the task.

### Word recall task

This test assesses short-term working memory. Participants were given three chances to recall as many words as possible from a list of ten words. The words were read aloud each time in a different order (MEM I, MEM II, MEM III). Each word recalled was graded with one point, maximum 10 points for each task.

### Word recognition task

The test measures the ability to recognize information. The participants were asked to read and remember a list of twelve words. They were then presented together with an other twelve new words and asked which words were seen previously. Scoring: one point for each correctly remembered word (maximum 12), and one point for each incorrectly recognized word, also max 12 words.

### Constructional praxis

This test measures visuospatial abilities. A sequence of four shapes, in an ascending order of difficulty, was presented to the participants who were asked to draw them, in two attempts. Scoring: all four shapes drawn correctly scored 0, one shape drawn incorrectly scored 1 point, two shapes drawn incorrectly scored 2 and so on till 5 which meant no clear shapes drawn.

### Naming objects and fingers

Participants were asked to name twelve real objects that were shown to them and state the name of each of the fingers of the dominant hand. Scoring: each object or finger correctly named was graded one point, to a maximum total of 17 points.

### Ideational praxis (following commands)

Women were asked to follow five simple and multi-step directions. Scoring: each correctly executed command scored one point. Maximum score: 5.

### Depression test

Women were given fifteen items taken from the Geriatric Depression Scale as this scale was validated for young and middle-aged adults as well^32^. The women were asked to rate the items by a “yes/no” response. Ten items indicate the presence of depression when answered positively, while the rest indicate depression when answered negatively Scoring: 0–4 no depression, 5–8 mild depression. 9–11 moderate and 12–15 severe depression.

### Statistical analysis

Statistical analysis was performed using SPSS software v23 (IBM Inc., USA). Normality of distribution was tested by the Kolmogorov–Smirnov test. All continuous variables deviated significantly from normal distribution, except for patient’s age. Comparisons between non-parametric variables were performed using the Mann–Whitney U-test. Comparisons between patients' age were performed using Student’s *t*-test. Nominal variables were compared using the chi-squared test or Fisher exact test, as appropriate. All tests were considered significant at *p* < 0.05. Multivariate linear regression analyses were performed to test the association between the study groups and cognitive subtests, after controlling for confounders. Age and number of years of education were controlled for, as these factors may affect cognitive tests results. Pregnancy was coded as a dichotomic variable (yes/no). Age and number of years of education were coded as continuous variables.

### Ethical approval

All procedures involving human participants in this study were in accordance with the ethical standards of the E. Wolfson Medical Center research committee and with the 1964 Helsinki declaration and its later amendments. Number of Helsinki committee approval 0216-16-WOMC.

### Informed consent

Informed consent was obtained from all individual participants included in the study.

## Results

Selected demographic and baseline characteristics are presented in Table 1. Mean age was similar between pregnant and non-pregnant women. Mean number of years of education and the rate of smoking were higher among non-pregnant women. Among pregnant women there were four suffering from hypothyroidism and two from hypertension and in the nonpregnant there were four with hypothyroidism, one with hypertension, two with asthma, one with lupus erithematosus and one with Crohn's disease.

**Table 1:** Selected women characteristics according to the study groups.

Characteristics	Pregnant (n = 40)	Non-pregnant (n = 40)	*p* value
Age, years	31.9 ± 4.1	31.7 ± 5.2	0.87
Gestational age	31.3 ± 6.3		
Number of years of education	15 (12–20)	17 (12–22)	0.005
Smoking	2 (5.0)	10 (25.0)	0.012
Medications	6 (15.0)	8 (20.0)	0.55
Diseases	6 (15.0)	9 (22.5)	0.39

Data are presented as mean ± SD, median (range), or n (%).

The results of the memory and cognitive tests are presented in Table 2. The performance scores on the difficult items of Verbal Paired Associates, the Digit Span forwards, and the Naming Objects and Fingers, were better in non-pregnant women. The performance scores for the easy items of the Verbal Paired Associates, Digit Span backwards, Trail Making Score part A and B, Ideational Praxis, Constructional Praxis,Word Recall, and Word Recognition were similar between pregnant and non-pregnant women. Constructional Praxis was reported as number (percent) of women who performed correctly (copyed 4 correct shapes and scored 0), while the rest made only one mistake and scored 1). None of the women were depressed and no difference was found on the depression test between pregnant and non-pregnant women.

**Table 2:** Results of cognition and memory test according to the study groups.

Characteristics	Pregnant (n = 40)	Non-pregnant (n = 40)	*p* value
**Verbal paired associates**			
Easy	12 (8–12)	12 (9–12)	0.064
Difficult	8 (2–12)	10 (3–12)	0.010
Total	20 (13–24)	22 (15–24)	0.009
**Digit span**			
Forward	10 (5–12)	11 (7–12)	0.004
Backwards	8 (5–12)	10 (6–12)	0.076
Total	17 (11–24)	20 (14–24)	0.010
**Trail Making Score (TMS)**			
TMS 1	26.9 (14.6–54.0)	27.5 (15.0–53.0)	0.81
TMS 2	57.3 (1.2–221.5)	54.8 (33.1–91.9)	0.22
Depression test	1 (0–9)	1 (0–8)	0.96
**Word Recall tests (MEM)**			
MEM1	7.5 (4–10)	8.0 (4–9)	0.56
MEM2	10 (6–10)	10 (7–10)	0.49
MEM3	10 (8–10)	10 (8–10)	0.32
Naming objects and fingers	17 (11–17)	17 (17–17)	< 0.001
Ideational praxis	5 (5–5)	5 (5–5)	1.0
Constructional praxis^a^	(30) 75%	(35) 87.5%	0.15
**Word recognition**			
Right	12 (9–12)	11 (9–12)	0.25
Wrong	0 (0–3)	1 (0–4)	0.10

^a^Data are presented as median (range) n (%).

The results of the multivariate linear regression analysis are presented in Table 3. After adjusting for age and number of years of education, pregnancy was still associated with lower Verbal Paired Associates score (beta − 2.0, 95% CI − 2.4 to − 0.008, *p* = 0.04), and lower Naming Objects and Fingers score (beta − 2.4, 95% CI − 1 to 0.09, *p* = 0.01), but the significance of the relationship with lower Digit Span was lost (beta 1.6, 95% CI − 2.7 to 0.24, *p* = 0.09).

**Table 3:** Multivariate linear regression analysis testing the association between pregnancy and different cognitive tests.

Test	Beta (95% confidence interval)	*p* value^a^
Verbal paired associates total	− 2.0 (− 2.4 to − 0.008)	0.04
Naming objects and fingers	− 2.4 (− 1.0 to − 0.09)	0.01
Digit span total	− 1.6 (− 2.7 to 0.24)	0.09

^a^Adjusted for age and numbers of years of education.

## Discussion

We found higher performance scores in the difficult items of Verbal Paired Associates, and the Naming Objects and Fingers, also a trending difference in the Digit Span forwards among non-pregnant women. These findings seem to suggest involvement of the lateral, medio-frontoparietal and occipital regions, prefrontal lobes and inferior left parietal lobule respectively. The test results demonstrate deficits in learning and memory tasks, as well as in attention and language abilities during pregnancy, thus reflecting a diffuse effect on the brain. This could be explained by the detrimental effects of hydrocortisone on acquisition and consolidation of information, as previously reported in the literature, in a group of young men^33^. There is also evidence that cognitive functions are affected by the increased levels of sex hormones that occur during pregnancy, mainly during the second and third trimester. Our patients were all recruited during the 2nd–3rd trimester as it was found that during the first trimester the plasma hormones may not reach the level required to affect cognitive processes^34,35^.

Our findings are supported by another study that reports a significantly lower memory encoding and retrieval in pregnant women than in a control group^15^.

Memory loss at such a critical period in a woman's life was speculated to result from hormonal changes or from a lifestyle shift, reflecting deeper concern regarding the delivery, the post-natal period, adaptation strategies for a new mother, sleep pattern changes, which may all cause possible anxiety or depression. A recent study reported a diminished memory function in a specific subset of pregnant women who display depressive symptoms associated with pregnancy^36^. However, in our study we did not find significant differences in the depression scores among pregnant versus non-pregnant women. This implies that hormonal changes represent the main culprit responsible for the cognitive decline, as already reported in previous studies^34,35^.

A recently published review of cognitive changes in pregnancy and postpartum that analyzed a substantial number of studies in animals and humans, found that there is little agreement on the type and the degree of memory impairment^37^. Cognitive performance seems to be diminished rather than enhanced in humans, whereas animal research overwhelmingly supports enhanced memory function during pregnancy. Glynn postulated that these different findings may reflect the differences between species or the different memory tests used in animals as opposed to human studies^38^. Our study examined more cognitive tasks than memory alone. Beyond the decline in memory we also found a deficit in language tasks.

To the best of our knowledge, our study is the first to report language performance among pregnant women. We found a lower performance in the Naming Objects and Fingers test among pregnant compared to the non-pregnant women. This finding reflects additional involvement of the dominant hemisphere speech areas.

The small clinical differences found in the domains of memory and language reflect the change in cognitive performance during pregnancy. Obviously, these differences do not reflect dementia, but subtle cognitive changes that occur during pregnancy and resolve a few months after delivery. These mild differences are not always clinically evident, unless searched for, using specific cognitive tests.

Interestingly, this is supported by brain imaging studies that show gray matter changes in pregnant women, demonstrating an adaptive behavior to the future role of the mother. In a recent study, brain MRI scans in women before becoming pregnant and again after completion of their first pregnancy, showed changes in the grey matter, and specifically a volume reduction. The hippocampus, which is associated with memory, had also shrunk during this period. These changes remained at the end of the 2-year study. No such changes were observed in the future fathers' brains^39^. The reproduction-related brain plasticity of pregnant women involves areas implicated in maternal caregiving, reward/motivation, salience/threat detection, emotional regulation and social cognition, such as the ability to empathize and infer the mental state of the baby^40^.

If at first glance, it is surprising that the brain would shrink during pregnancy, causing the so-called "pregnancy fog" characterized by some type of forgetfulness, the answer is logical. We hypothesize that since the brain lies in a limited space and closed box, this prior shrinkage is required in order to prepare it for further development after parturition. The mother-baby bonding requires lots of adaptive changes in the postpartum mother's brain, which are linked to areas concerned with social cognition, primarily in networks involved in motivation, somatosensory information and executive functions. These may be then mediated by experience-dependent plasticity during the postpartum period. We consider the possibility that apparent deficits in some cognitive areas during pregnancy and the postpartum period reflect a trade-off; whereby essential cognitive functions that are required by women during the reproductive phase are given precedence. This hypothesis is supported by the results of our study as most cognitive functions examined did not, in fact, decline. After adjusting for education and age, the attention task reflected by the Digit Span forward test, was similar between pregnant and non-pregnant women. The performance on attention, working memory, and praxis related items did not decline in pregnant women, highlighting a cognitive reorganization of the brain that might suggest enhanced social cognition, including facilitated nonverbal processing of emotions, directed towards establishing a bond between baby and mother^24^.

The strength of our study is related to the multiple cognitive tests used for several domains, in addition to memory testing. Furthermore, we examined language abilities in pregnant women, a feature that was not previously reported.

The limitations include a possible selection bias due to a non-randomized recruitment and testing only at one point in time each woman, without taking into account the differences that might appear during a repeated test. An additional limitation is the possibility of an alfa inflation (type I error) by performing multiple tests.

## Conclusion

Our study demonstrates a significant verbal memory and learning deficit in late pregnancy and a language deficit (in naming), which is a novel finding that has not been previously reported.
